# Salvage resection for FGFR2 fusion-positive intrahepatic cholangiocarcinoma after pemigatinib therapy: a case report and literature review

**DOI:** 10.3389/fonc.2026.1798918

**Published:** 2026-07-08

**Authors:** Yuhang Kang, Fei Xie, Zhenru Wu, Yongjie Zhou, Tianfu Wen, Xiaoyun Zhang

**Affiliations:** 1 Division of Liver Surgery, Department of General Surgery, West China Hospital, Sichuan University, Chengdu, China; 2 Department of Hepatic-Biliary-Pancreatic Surgery, the First People’s Hospital of Neijiang, Neijiang, China; 3 Institute of Clinical Pathology, West China Hospital, Sichuan University, Chengdu, China; 4 Laboratory of Liver Transplantation, Key Laboratory of Transplant Engineering and Immunology, National Health Commission (NHC), West China Hospital, Sichuan University, Chengdu, China

**Keywords:** FGFR2, intrahepatic cholangiocarcinoma, pemigatinib, salvage resection, targeted therapy

## Abstract

**Background:**

The incidence of intrahepatic cholangiocarcinoma (iCCA) is rising. Unfortunately, most patients present with locally advanced or metastatic disease at initial diagnosis, making them ineligible for surgical resection. Currently, the standard first-line treatment for unresectable iCCA is gemcitabine plus cisplatin combined with durvalumab or pembrolizumab, but the survival benefit remains limited. Pemigatinib is recommended as the treatment of previously treated advanced iCCA with FGFR2 fusion or rearrangement. The favorable objective response rate in FIGHT-302 study highlights its potential in salvage resection following tumor shrinkage and downstaging. Salvage resection has been implemented in various initially unresectable malignancies, significantly improving patient prognosis. To date, few reports have described salvage resection following FGFR inhibitor therapy for FGFR2 fusion-positive iCCA.

**Case presentation:**

A 66-year-old female diagnosed with locally advanced iCCA harboring *FGFR2* fusion received 5 cycles of pemigatinib treatment after a multidisciplinary team discussion. The efficacy was assessed as partial remission per the Response Evaluation Criteria in Solid Tumors version 1.1, and the patient underwent a laparoscopic left hepatectomy guided by indocyanine green fluorescence. During the treatment, there was no evidence of severe adverse events and postoperative complications. The patient remained recurrence-free at the last follow-up of 30 months postoperatively.

**Conclusion:**

This is the first report of salvage resection following first-line pemigatinib monotherapy for *FGFR2* fusion-positive locally advanced iCCA, with promising efficacy and acceptable safety.

## 1 Introduction

Intrahepatic cholangiocarcinoma (iCCA) accounts for 10%–15% of primary liver cancers and is distinguished by insidious onset, rapid progression, and poor prognosis (1–3). Despite its rarity, the incidence is rising globally (4, 5). Most patients present with locally advanced or metastatic disease at diagnosis, with <30% eligible for curative surgical resection (6). Even for resectable cases, the risk of recurrence remains high, and the 5-year overall survival (OS) rate is merely 20–35%, highlighting an urgent unmet need for novel therapeutic strategies in unresectable iCCA (7, 8).

Advances in genomic profiling have identified multiple actionable molecular alterations in iCCA. The fibroblast growth factor receptor (*FGFR*) 2 fusions or rearrangements are among the most well-characterized, occurring in 10–15% of iCCA and largely mutually exclusive with IDH1 mutations (9–11). These fusions drive tumor proliferation, angiogenesis, and invasion via constitutive activation of the *FGFR* signaling pathway, making them ideal therapeutic targets (10, 12). Pemigatinib is an oral, potent, and selective inhibitor of *FGFR1*, *FGFR2*, and *FGFR3* (13). Owing to the success of FIGHT-202 study, it has been approved by regulatory authorities in the United States, European Union, Japan, and China for the treatment of previously treated advanced cholangiocarcinoma harboring *FGFR2* fusion or rearrangement (14). Moreover, the phase III FIGHT-302 trial (NCT03656536) was designed to evaluate first line pemigatinib versus gemcitabine plus cisplatin in previously untreated patients with FGFR2-rearranged unresectable cholangiocarcinoma (15). Recent results presented at American Society of Clinical Oncology (ASCO) 2026 demonstrated a significant efficacy (16).

To date, few reports have described salvage resection following *FGFR* inhibitor therapy for *FGFR2* fusion-positive iCCA. Herein, we present a case of locally advanced iCCA that underwent successful salvage laparoscopic hepatectomy with indocyanine green (ICG) fluorescence guidance after first-line pemigatinib treatment, and review relevant literature to discuss the clinical value of this approach.

## 2 Case presentation

**Figure 1:**
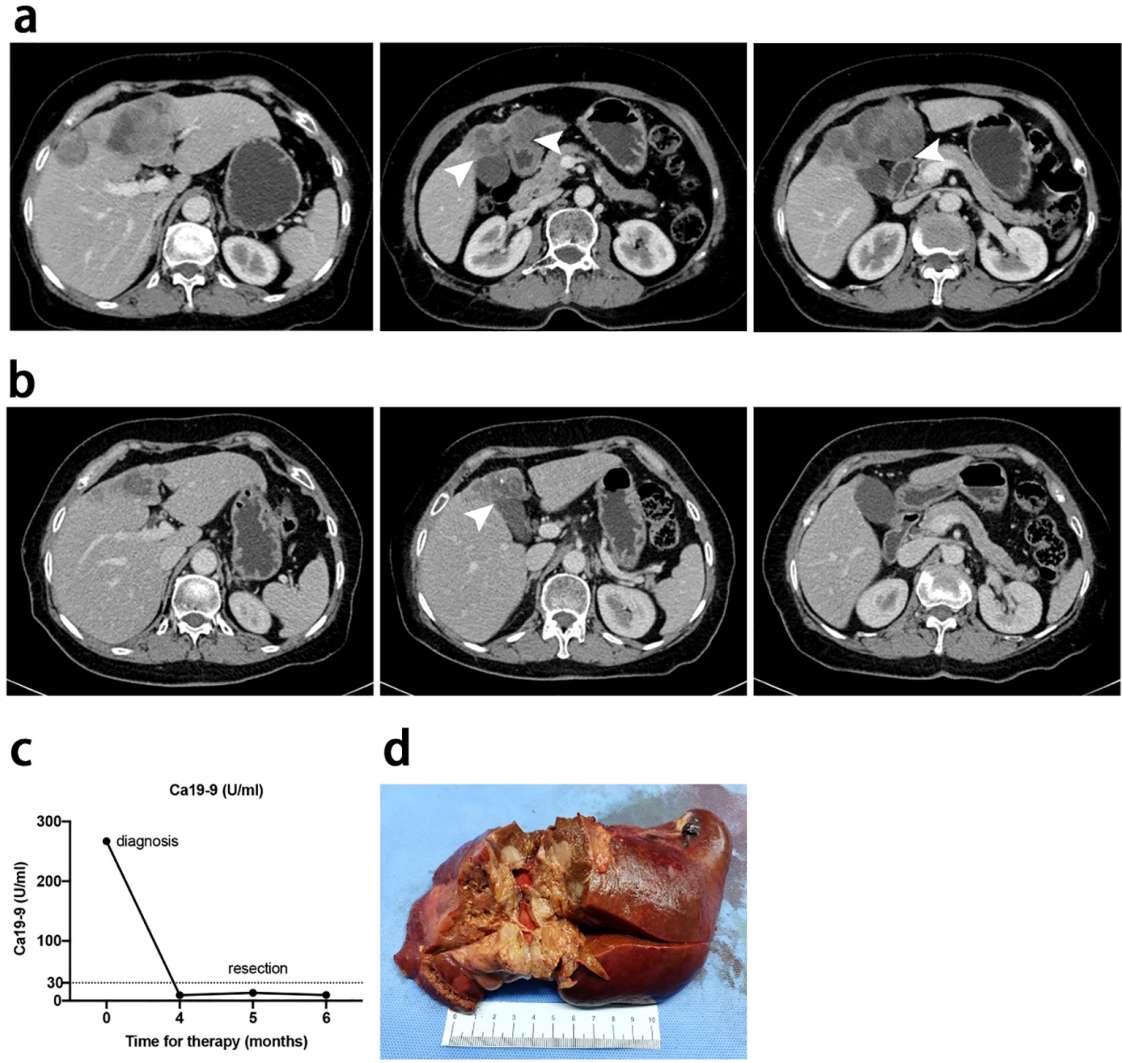
Salvage resection for FGFR2 fusion-positive intrahepatic cholangiocarcinoma after pemigatinib therapy **(a)** before pemigatinib therapy, CT showed that the largest diameter of the tumor at left liver was 9.76 cm, and the stomach, duodenum and gallbladder are suspected to be invaded (white arrow); **(b)** after pemigatinib therapy, tumor has significantly shrunk and the gallbladder is suspected to be invaded (white arrow); **(c)** CA19–9 level of the patient; **(d)** specimen of the tumor.

**Figure 2:**
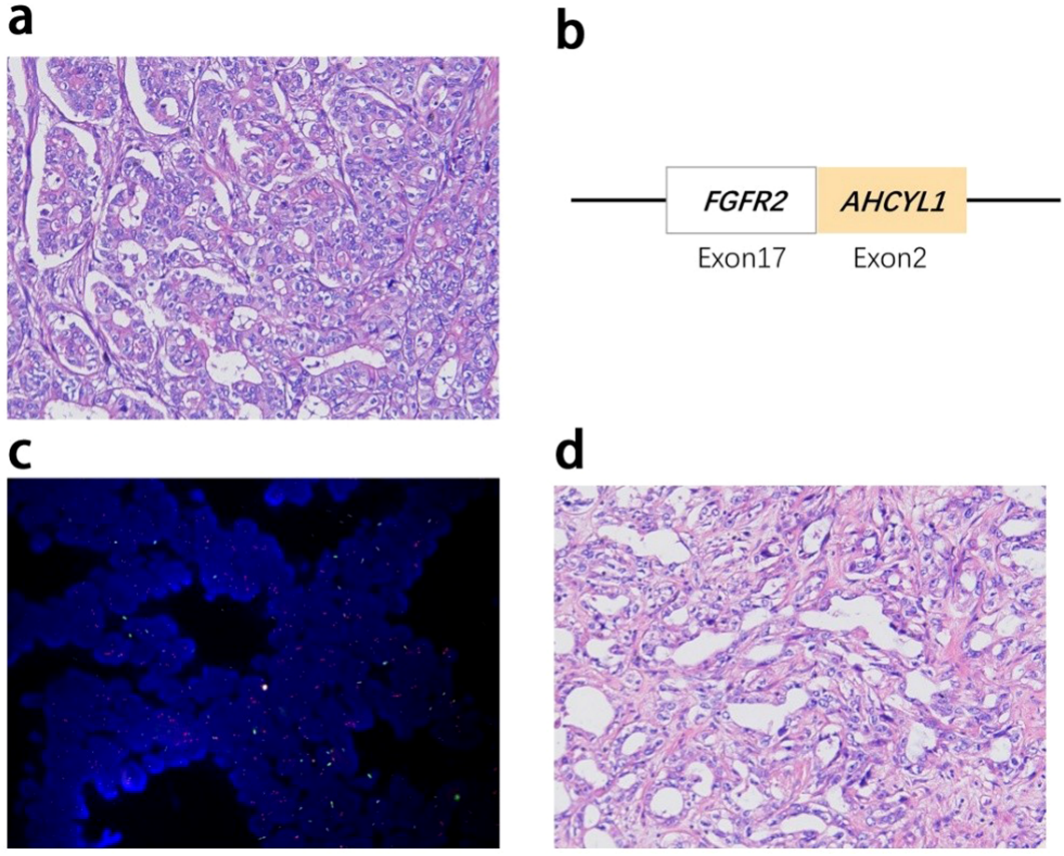
Pathology, next-generation sequencing and fluorescence *in situ* hybridization of intrahepatic cholangiocarcinoma. **(a)** hematoxylin eosin (HE) staining of cholangiocarcinoma before pemigatinib therapy; **(b)** The result of NGS (FoundationOne^®^, Foundation Medicine Inc., MA, USA) is *FGFR2-AHCYL1* fusion; **(c)** FGFR2 gene breakage was positive by fluorescence *in situ* hybridization; **(d)** HE-staining after resection.

A 66-year-old female presented to West China Hospital of Sichuan University with 2 weeks of right upper abdominal discomfort. She had a history of resolved prior hepatitis B virus (HBV) exposure with negative hepatitis B surface antigen (HBsAg) and undetectable HBV DNA. The serum tumor marker CA19–9 was significantly elevated. The Eastern Cooperative Oncology Group (ECOG) performance status was 0, and liver function was Child-Pugh class A (5 points). Abdominal contrast-enhanced computed tomography (CT) revealed a large irregular mass in the left hepatic lobe with heterogeneous enhancement and a maximum diameter of 9.76 cm, which was suspected to invade the stomach, duodenum, and gallbladder, without distant metastases (**Figure 1a**). Ultrasound-guided liver biopsy confirmed adenocarcinoma consistent with cholangiocarcinoma (**Figure 2a**). Based on the 8th edition of the American Joint Committee on Cancer (AJCC) staging system, the patient was diagnosed with locally advanced iCCA (T4N0M0).

Next-generation sequencing (NGS) of the biopsy tissue identified an *FGFR2-AHCYL1* fusion (**Figure 2b**), which was further confirmed by fluorescence *in situ* hybridization (FISH) demonstrating *FGFR2* gene breakage (**Figure 2c**). Neither isocitrate dehydrogenase 1 (IDH1) codon 132 nor IDH2 codon 172 mutation was detected.

Following a multidisciplinary team (MDT) discussion for primary liver cancer, the patient was formally enrolled in the FIGHT-302 trial (Ethics Approval Number: 2020-235) and received first-line pemigatinib (13.5 mg once daily, continuous 3-week cycle) due to her locally advanced unresectable disease and *FGFR2* fusion positivity. Adverse events were graded per Common Terminology Criteria for Adverse Events version 5.0 (CTCAE v5.0) and managed symptomatically, including hyperphosphatemia (Grade 2), diarrhea, alopecia, hand-foot syndrome, blurred vision, and oral mucositis (all Grade 1), without treatment interruption or dose modification.

The first protocol-mandated tumor re-evaluation after Cycle 3 confirmed partial remission (PR) per Response Evaluation Criteria in Solid Tumors version 1.1 (RECIST v1.1) with normalization of serum CA19-9. At the follow-up after Cycle 5, the patient expressed a preference for surgical resection. An additional abdominal CT showed further tumor shrinkage, with the maximum diameter reduced to 4.85 cm and no evidence of extrahepatic invasion (**Figure 1b**). The serum CA19–9 level remained normal (**Figure 1c**).

**Figure 3:**
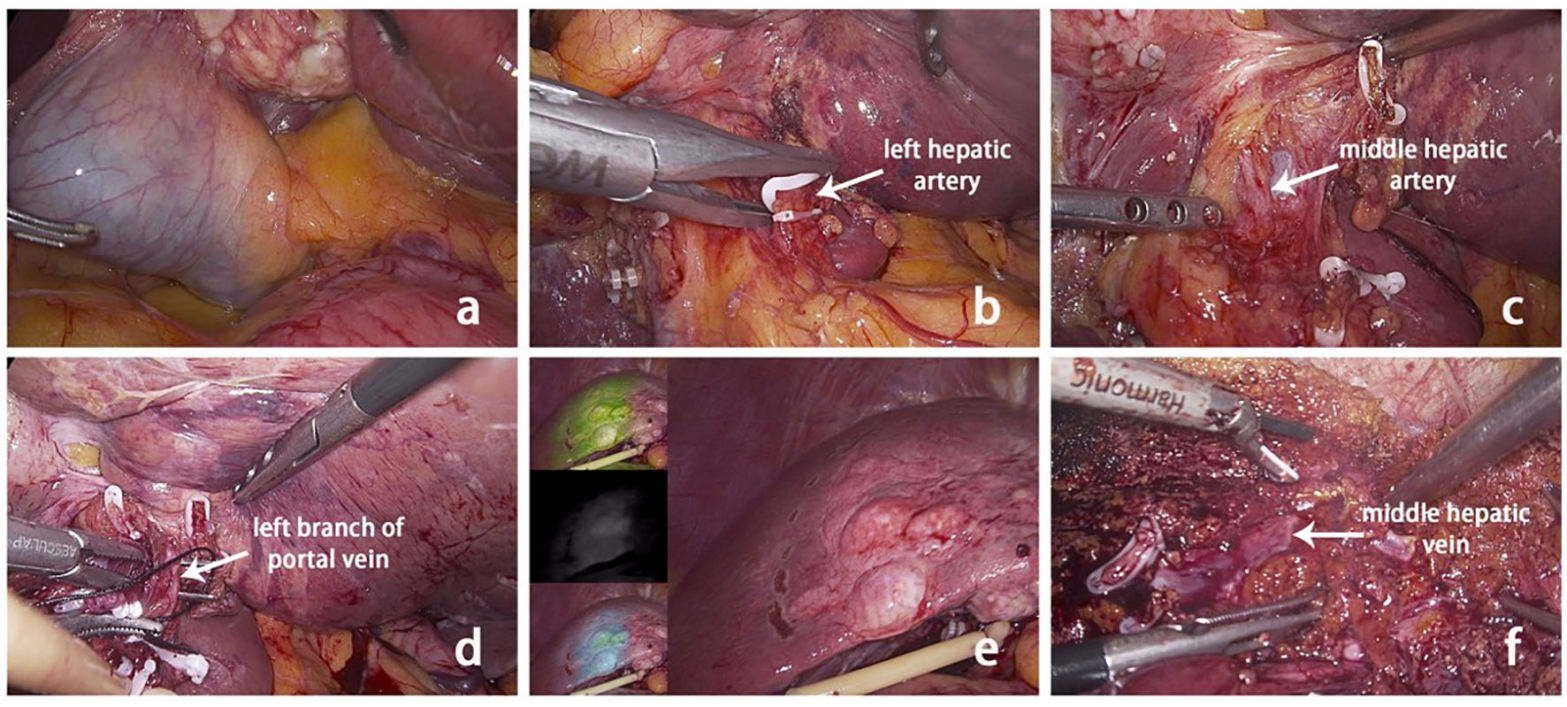
Laparoscopic hepatectomy guided by indocyanine green fluorescence (ICG)-fluorescence imaging. We planned to perform laparoscopic anatomical left hepatectomy using negative staining technique with ICG-fluorescence imaging. **(a)** no gastric and duodenal invasion was found during laparoscopic exploration; **(b)** ligation the left hepatic artery; **(c)** ligation the middle hepatic artery; **(d)** ligation of left portal vein; **(e)** negative staining shows that the tumor exceeded the left lobe of liver and extended left hepatectomy was performed; **(f)** the middle hepatic vein (MHV) was exposed adequately, and the liver parenchyma was dissected along the left side of the MHV until the left liver was resected.

Given the significant tumor downstaging, the MDT reassessed resectability and recommended salvage surgical resection. Two weeks after the final pemigatinib dose, the patient underwent laparoscopic exploration. Intraoperative indocyanine green (ICG) fluorescence imaging (0.5 mg/kg ICG administered intravenously 24 hours preoperatively) was used to delineate the tumor boundary (**Figure 3**), and a left hepatectomy was performed successfully (**Figure 1d**).

Postoperative recovery was uneventful, with no major complications (e.g., bile leakage, hemorrhage, liver failure), and the patient was discharged on postoperative day 7. Postoperative pathology confirmed no vascular or lymphatic invasion and negative surgical margins (R0 resection) (**Figure 2d**). Immunohistochemistry demonstrated proficient mismatch repair (pMMR) status (MLH1+, MSH2+, MSH6+, PMS2+) and HER2 negativity (score 0). Treatment-related fibrotic and hyalinized changes comprised approximately 20% of the specimen, consistent with partial pathological response. No adjuvant therapy was administered given the lack of evidence in this setting, and regular follow-up was performed every 3 months for the first 2 years, then every 6 months thereafter. At the 30-month postoperative follow-up, no radiological evidence of recurrence was observed, and the serum CA19–9 level remained normal.

## 3 Discussion

The global incidence of iCCA is projected to increase by up to 10-fold over the next 20–30 years (17). Surgical resection remains the only curative treatment for iCCA, but its insidious onset, diagnostic challenges, and high invasiveness result in most patients presenting with unresectable disease at initial diagnosis. For over a decade, systemic chemotherapy has been the mainstay of treatment for these patients. Based on the ABC-02 trial, gemcitabine plus cisplatin is the standard first-line regimen for locally advanced and metastatic cholangiocarcinoma, with a median OS of 11.7 months (18).

Recently, two pivotal trials have improved outcomes for unresectable iCCA by combining standard chemotherapy with immunotherapy. The TOPAZ-1 trial showed that gemcitabine, cisplatin, and durvalumab prolonged median OS to 12.9 months, while the KEYNOTE-966 trial demonstrated an improved median OS of 12.7 months with gemcitabine, cisplatin, and pembrolizumab compared to chemotherapy alone (19, 20). Consequently, both the National Comprehensive Cancer Network (NCCN) and European Society for Medical Oncology (ESMO) recommend durvalumab or pembrolizumab plus gemcitabine and cisplatin as first-line treatment for unresectable iCCA (21, 22). Despite these advances, treatment options remain limited, and overall efficacy remains suboptimal.

Salvage resection aims to achieve radical resection of initially unresectable tumors that shrink or downstage after multimodal therapy including chemotherapy, targeted therapy, immunotherapy and locoregional therapy, thereby improving long-term survival. First described in 1970 for a patient with a large hepatoblastoma following radiotherapy and chemotherapy, salvage resection has since been successfully implemented in unresectable hepatocellular carcinoma and other malignancies (e.g., colorectal, gastric, pancreatic cancer), conferring significant survival benefits (23–28). For iCCA, salvage resection experience was historically limited due to the lack of effective antitumor therapies, with only sporadic reports in patients receiving systemic chemotherapy or transarterial chemoembolization (29, 30). In recent years, targeted therapies and immunotherapies have increased surgical eligibility and improved postoperative survival (31–37) (**Table 1**). Although prospective data are lacking, retrospective studies suggest favorable prognoses following salvage resection, supporting its role as an important strategy for initially unresectable iCCA (38, 39).

**Table 1:** Representative preoperative treatment for salvage resection of unresectable iCCA.

Study design	Treatment	Sample size (n)	Conversion rate (%)	Reference
Clinical trial	Toripalimab + lenvatinib + Gemox	30	10	Shi et al (31), 2023
Clinical trial	GC+DEBIRI	24	25 (downsizing to resection or ablation)	Martin et al (32), 2022
Retrospective study	TACE + lenvatinib	44	63.6	Yuan et al (33), 2022
Retrospective study	Gemox-HAIC + Gem-SYS + lenvatinib + PD-1 inhibitor	21	19	Ni et al (34), 2024
Retrospective study	HAIC + TKIs + anti-PD-1	39	15.4	Zhang et al (35), 2022
Case report	GC + pembrolizumab	1	–	Gonzalez et al (36), 2026
Case report	Durvalumab + Gemox + lenvatinib	1	–	Tan et al (37), 2025

iCCA, intrahepatic cholangiocarcinoma; Gemox, gemcitabine and oxaliplatin; GC, gemcitabine plus cisplatin; DEBIRI, irinotecan drug-eluting beads; TACE, transarterial chemoembolization; HAIC, hepatic arterial infusion chemotherapy; Gem-SYS, systemic gemcitabine chemotherapy; PD-1, programmed cell death protein 1; TKIs, tyrosine kinase inhibitors.

Precision medicine developed for promising molecular targets is becoming a reality (1, 40). Several targeted agents are recommended for unresectable cholangiocarcinoma harboring *NTRK* fusions, *MSI-H/dMMR*, *TMB-H*, or *RET* fusions (21). For *FGFR2* fusion-positive iCCA, pemigatinib is recommended as second-line treatment for post-chemotherapy progression, based on a median OS of 17.5 months in the FIGHT-202 study (41). Building on promising phase 2 results, pemigatinib was evaluated as first-line therapy in the phase 3 FIGHT-302 trial, which compared pemigatinib monotherapy to gemcitabine plus cisplatin (15). Although FIGHT-302 was terminated early due to changes in the standard first-line treatment, its results presented at ASCO 2026 confirmed significant first-line efficacy of pemigatinib in FGFR2-rearranged cholangiocarcinoma, with an objective response rate of 47.0%, a median duration of response of 14.2 months, and a median progression-free survival of 8.3 months, supporting its potential to achieve salvage resection in selected patients (16). Kaneko, J. et al. reported a case of initially unresectable iCCA that progressed after chemotherapy and achieved successful salvage resection following second-line pemigatinib administration, which preliminarily demonstrates the efficacy of pemigatinib in salvage resection (42). Yang et al. described conversion surgery in a patient with recurrent FGFR2 fusion-positive iCCA following pemigatinib combined with sintilimab, with no recurrence at 19 months postoperatively (43).

To the best of our knowledge, we provide the first report of salvage resection following first-line pemigatinib monotherapy for *FGFR2* fusion-positive locally advanced iCCA. In contrast to the complete radiographic response achieved in the transplantation case reported by Byrne et al., our patient achieved partial response (44). Considering R0 resectability, donor organ accessibility, and financial constraints, the MDT opted for surgical resection over liver transplantation, with the patient achieving 30-month recurrence-free survival. Postoperative pathology revealed partial rather than complete response. The prognostic value of this finding in the context of FGFR-targeted conversion therapy remains uncertain, and long-term outcomes require continued follow-up. Additionally, ctDNA-based minimal residual disease monitoring was not performed due to limitations in treatment protocol and accessibility. Further high-quality evidence from prospective studies is needed to establish the role of FGFR-targeted conversion therapy.

The optimal timing of salvage resection is unclear. The median progression-free survival was 8.3 months for patients with *FGFR2* fusion according to FIGHT-302 trial (16). Therefore, we suggest that the operation time should be within 8 months to avoid acquired resistance mutations caused by clonal evolution during *FGFR* inhibitor treatment (45). Notably, salvage resection for unresectable iCCA remains exploratory, involving critical steps including resectability assessment, comprehensive molecular testing, multimodal therapy administration, accurate radiological re-evaluation, and individualized surgical planning. Thus, MDT involvement (hepatobiliary surgeons, medical oncologists, radiologists, pathologists, interventional radiologists, gastroenterologists) is paramount. This case may serve as a reference for MDT management of unresectable iCCA (46).

Several ongoing trials are investigating novel combination therapies for advanced iCCA (34, 47–49). For *FGFR2* fusion-positive iCCA, neither TOPAZ-1 nor KEYNOTE-966 reported efficacy outcomes specific to this molecular subgroup, leaving the benefit of ICI-based chemoimmunotherapy in terms of conversion surgery and survival unclear. Given the encouraging results of FIGHT-302, the survival benefits of pemigatinib combined with chemotherapy, interventional therapy, or immunotherapy warrant further prospective investigation.

## Data Availability

The raw data supporting the conclusions of this article will be made available by the authors, without undue reservation.

## References

[1] Valle JW Lamarca A Goyal L Barriuso J Zhu AX . New horizons for precision medicine in biliary tract cancers. Cancer Discov. (2017) 7:943–62. doi: 10.1158/2159-8290.Cd-17-0245 28818953 PMC5586506

[2] Florio AA Ferlay J Znaor A Ruggieri D Alvarez CS Laversanne M . Global trends in intrahepatic and extrahepatic cholangiocarcinoma incidence from 1993 to 2012. Cancer. (2020) 126:2666–78. doi: 10.1002/cncr.32803 32129902 PMC7323858

[3] Bray F Laversanne M Sung H Ferlay J Siegel RL Soerjomataram I . Global cancer statistics 2022: Globocan estimates of incidence and mortality worldwide for 36 cancers in 185 countries. CA Cancer J Clin. (2024) 74:229–63. doi: 10.3322/caac.21834 38572751

[4] Khan SA Tavolari S Brandi G . Cholangiocarcinoma: Epidemiology and risk factors. Liver Int. (2019) 39:19–31. doi: 10.1111/liv.14095 30851228

[5] Wang Y Alsaraf Y Bandaru SS Lyons S Reap L Ngo T . Epidemiology, survival and new treatment modalities for intrahepatic cholangiocarcinoma. J Gastrointest Oncol. (2024) 15:1777–88. doi: 10.21037/jgo-24-165 39279977 PMC11399825

[6] Banales JM Marin JJG Lamarca A Rodrigues PM Khan SA Roberts LR . Cholangiocarcinoma 2020: The next horizon in mechanisms and management. Nat Rev Gastroenterol Hepatol. (2020) 17:557–88. doi: 10.1038/s41575-020-0310-z 32606456 PMC7447603

[7] Mavros MN Economopoulos KP Alexiou VG Pawlik TM . Treatment and prognosis for patients with intrahepatic cholangiocarcinoma: Systematic review and meta-analysis. JAMA Surg. (2014) 149:565–74. doi: 10.1001/jamasurg.2013.5137 24718873

[8] Sirica AE Gores GJ Groopman JD Selaru FM Strazzabosco M Wei Wang X . Intrahepatic cholangiocarcinoma: Continuing challenges and translational advances. Hepatology. (2019) 69:1803–15. doi: 10.1002/hep.30289 30251463 PMC6433548

[9] Arai Y Totoki Y Hosoda F Shirota T Hama N Nakamura H . Fibroblast growth factor receptor 2 tyrosine kinase fusions define a unique molecular subtype of cholangiocarcinoma. Hepatology. (2014) 59:1427–34. doi: 10.1002/hep.26890 24122810

[10] Lamarca A Barriuso J McNamara MG Valle JW . Molecular targeted therapies: Ready for "prime time" in biliary tract cancer. J Hepatol. (2020) 73:170–85. doi: 10.1016/j.jhep.2020.03.007 32171892

[11] Vogel A Segatto O Stenzinger A Saborowski A . Fgfr2 inhibition in cholangiocarcinoma. Annu Rev Med. (2023) 74:293–306. doi: 10.1146/annurev-med-042921-024707 36170665

[12] Borad MJ Champion MD Egan JB Liang WS Fonseca R Bryce AH . Integrated genomic characterization reveals novel, therapeutically relevant drug targets in fgfr and egfr pathways in sporadic intrahepatic cholangiocarcinoma. PloS Genet. (2014) 10:e1004135. doi: 10.1371/journal.pgen.1004135 24550739 PMC3923676

[13] Liu PC Wu L Koblish H Bowman K Zhang Y Klabe R . Abstract 771: Preclinical characterization of the selective fgfr inhibitor incb054828. Cancer Res. (2015) 75:771. doi: 10.1158/1538-7445.Am2015-771 36230740

[14] Abou-Alfa GK Sahai V Hollebecque A Vaccaro G Melisi D Al-Rajabi R . Pemigatinib for previously treated, locally advanced or metastatic cholangiocarcinoma: A multicentre, open-label, phase 2 study. Lancet Oncol. (2020) 21:671–84. doi: 10.1016/s1470-2045(20)30109-1 32203698 PMC8461541

[15] Bekaii-Saab TS Valle JW Van Cutsem E Rimassa L Furuse J Ioka T . Fight-302: First-line pemigatinib vs gemcitabine plus cisplatin for advanced cholangiocarcinoma with fgfr2 rearrangements. Future Oncol. (2020) 16:2385–99. doi: 10.2217/fon-2020-0429 32677452 PMC9892961

[16] Bekaii-Saab TS Melisi D Wilmink H Garufi C Tran N Tortora G . Pemigatinib for unresectable or metastatic cholangiocarcinoma with fibroblast growth factor receptor-2 rearrangement: Results from the phase 3 fight-302 trial. J Clin Oncol. doi: 10.1200/jco-26-00788 42223137 PMC13641261

[17] Ilyas SI Khan SA Hallemeier CL Kelley RK Gores GJ . Cholangiocarcinoma - evolving concepts and therapeutic strategies. Nat Rev Clin Oncol. (2018) 15:95–111. doi: 10.1038/nrclinonc.2017.157 28994423 PMC5819599

[18] Valle J Wasan H Palmer DH Cunningham D Anthoney A Maraveyas A . Cisplatin plus gemcitabine versus gemcitabine for biliary tract cancer. N Engl J Med. (2010) 362:1273–81. doi: 10.1056/NEJMoa0908721 20375404

[19] Burris HA 3rd Okusaka T Vogel A Lee MA Takahashi H Breder V . Durvalumab plus gemcitabine and cisplatin in advanced biliary tract cancer (Topaz-1): Patient-reported outcomes from a randomised, double-blind, placebo-controlled, phase 3 trial. Lancet Oncol. (2024) 25:626–35. doi: 10.1016/s1470-2045(24)00082-2 38697156

[20] Kelley RK Ueno M Yoo C Finn RS Furuse J Ren Z . Pembrolizumab in combination with gemcitabine and cisplatin compared with gemcitabine and cisplatin alone for patients with advanced biliary tract cancer (Keynote-966): A randomised, double-blind, placebo-controlled, phase 3 trial. Lancet. (2023) 401:1853–65. doi: 10.1016/s0140-6736(23)00727-4 37075781

[21] Benson AB 3rd D'Angelica MI Abrams T Ahmed A Akce M Anaya DA . Biliary tract cancers, version 2.2025, nccn clinical practice guidelines in oncology. J Natl Compr Canc Netw. (2025) 23:403–18. doi: 10.6004/jnccn.2025.0042 40930144

[22] Vogel A Bridgewater J Edeline J Kelley RK Klümpen HJ Malka D . Biliary tract cancer: Esmo clinical practice guideline for diagnosis, treatment and follow-up. Ann Oncol. (2023) 34:127–40. doi: 10.1016/j.annonc.2022.10.506 36372281

[23] Hermann RE Lonsdale D . Chemotherapy, radiotherapy, and hepatic lobectomy for hepatoblastoma in an infant: Report of a survival. Surgery. (1970) 68:383–8. 4317928

[24] Singh P Hoffman K Schaverien MV Krause KJ Butler C Smith BD . Neoadjuvant radiotherapy to facilitate immediate breast reconstruction: A systematic review and current clinical trials. Ann Surg Oncol. (2019) 26:3312–20. doi: 10.1245/s10434-019-07538-x 31342362

[25] Chan KKK Saluja R Delos Santos K Lien K Shah K Cramarossa G . Neoadjuvant treatments for locally advanced, resectable esophageal cancer: A network meta-analysis. Int J Cancer. (2018) 143:430–7. doi: 10.1002/ijc.31312 29441562

[26] Lau WY Lai EC . Salvage surgery following downstaging of unresectable hepatocellular carcinoma--a strategy to increase resectability. Ann Surg Oncol. (2007) 14:3301–9. doi: 10.1245/s10434-007-9549-7 17891443

[27] Ludmir EB Palta M Willett CG Czito BG . Total neoadjuvant therapy for rectal cancer: An emerging option. Cancer. (2017) 123:1497–506. doi: 10.1002/cncr.30600 28295220

[28] Zhang X Cai H Peng W Wang H Wu J Zhu X . Lenvatinib plus transarterial chemoembolization and pd-1 inhibitors as conversion therapies for unresectable intermediate-advanced hepatocellular carcinoma: A phase 2 trial and exploratory biomolecular study. Signal Transduct Target Ther. (2026) 11:37. doi: 10.1038/s41392-025-02498-z 41565617 PMC12823698

[29] Tanaka N Yamakado K Nakatsuka A Fujii A Matsumura K Takeda K . Arterial chemoinfusion therapy through an implanted port system for patients with unresectable intrahepatic cholangiocarcinoma--initial experience. Eur J Radiol. (2002) 41:42–8. doi: 10.1016/s0720-048x(01)00414-4 11750151

[30] Wu Y Saiura A Yamamoto J Koga R Asahara S Kamei A . Locally advanced intrahepatic cholangiocarcinoma successfully resected after transcatheter arterial chemoembolization with degradable starch microspheres: Report of a case. Hepatogastroenterology. (2007) 54:1345–7. 17708251

[31] Shi GM Huang XY Wu D Sun HC Liang F Ji Y . Toripalimab combined with lenvatinib and gemox is a promising regimen as first-line treatment for advanced intrahepatic cholangiocarcinoma: A single-center, single-arm, phase 2 study. Signal Transduct Target Ther. (2023) 8:106. doi: 10.1038/s41392-023-01317-7 36928584 PMC10020443

[32] Martin RCG 2nd Simo KA Hansen P Rocha F Philips P McMasters KM . Drug-eluting bead, irinotecan therapy of unresectable intrahepatic cholangiocarcinoma (Deltic) with concomitant systemic gemcitabine and cisplatin. Ann Surg Oncol. (2022) 29:5462–73. doi: 10.1245/s10434-022-11932-3 35657463

[33] Yuan P Song J Wang F Zhu G Chen B . Combination of tace and lenvatinib as a promising option for downstaging to surgery of initially unresectable intrahepatic cholangiocarcinoma. Invest New Drugs. (2022) 40:1125–32. doi: 10.1007/s10637-022-01257-z 35793038

[34] Ni JY Sun HL Guo GF Zhou X Wei JX Xu LF . Hepatic arterial infusion of gemox plus systemic gemcitabine chemotherapy combined with lenvatinib and pd-1 inhibitor in large unresectable intrahepatic cholangiocarcinoma. Int Immunopharmacol. (2024) 140:112872. doi: 10.1016/j.intimp.2024.112872 39121605

[35] Zhang N Yu BR Wang YX Zhao YM Zhou JM Wang M . Clinical outcomes of hepatic arterial infusion chemotherapy combined with tyrosine kinase inhibitors and anti-pd-1 immunotherapy for unresectable intrahepatic cholangiocarcinoma. J Dig Dis. (2022) 23:535–45. doi: 10.1111/1751-2980.13127 36148493

[36] Gonzalez JFS Aliseda D Harrison JM Visser BC . Robotic left hepatectomy with hilar dissection and portal lymphadenectomy following preoperative gemcitabine, cisplatin, and pembrolizumab for intrahepatic cholangiocarcinoma. Ann Surg Oncol. (2026) 33:1536–7. doi: 10.1245/s10434-025-18515-y 41109879

[37] Tan W Wei J Chen Y Shang C . Laparoscopic extended segmentectomy 8 with right hepatic vein resection after conversion therapy for advanced intrahepatic cholangiocarcinoma. Ann Surg Oncol. (2025) 32:5711–2. doi: 10.1245/s10434-025-17392-9 40397343

[38] Wang S Wang Y Zhu C Liu K Chao J Zhang N . Conversion surgery intervention versus continued systemic therapy in patients with a response after pd-1/pd-l1 inhibitor-based combination therapy for initially unresectable biliary tract cancer: A retrospective cohort study. Int J Surg. (2024) 110:4608–16. doi: 10.1097/js9.0000000000001540 38704621 PMC11326034

[39] Zhou Y Wang Q Lin M Wang S . Survival benefit of conversion surgery for initially unresectable biliary tract cancer: A systematic review and meta-analysis. Langenbecks Arch Surg. (2025) 410:63. doi: 10.1007/s00423-025-03630-x 39918658 PMC11805778

[40] Dong L Lu D Chen R Lin Y Zhu H Zhang Z . Proteogenomic characterization identifies clinically relevant subgroups of intrahepatic cholangiocarcinoma. Cancer Cell. (2022) 40:70–87.e15. doi: 10.1016/j.ccell.2021.12.006 34971568

[41] Vogel A Sahai V Hollebecque A Vaccaro GM Melisi D Al Rajabi RM . An open-label study of pemigatinib in cholangiocarcinoma: Final results from fight-202. ESMO Open. (2024) 9:103488. doi: 10.1016/j.esmoop.2024.103488 38838500 PMC11190465

[42] Kaneko J Kiuchi R Takinami M Ohnishi I Ito J Jindo O . Successful intrahepatic cholangiocarcinoma conversion surgery after administration of fibroblast growth factor receptor inhibitor. Clin J Gastroenterol. (2024) 17:936–42. doi: 10.1007/s12328-024-02014-w 38985249 PMC11436442

[43] Yang Y Li J Ma D Hao F Li W Xie J . Systemic therapy with pemigatinib and sintilimab followed by resection for recurrent fgfr-2-positive intrahepatic cholangiocarcinoma: A case report. Front Oncol. (2025) 15:2025. doi: 10.3389/fonc.2025.1527372 40255433 PMC12006668

[44] Byrne MM Dunne RF Melaragno JI Chávez-Villa M Hezel A Liao X . Neoadjuvant pemigatinib as a bridge to living donor liver transplantation for intrahepatic cholangiocarcinoma with FGFR2 gene rearrangement. Am J Transplant. (2025) 25:623–7. doi: 10.1016/j.ajt.2024.10.023 39515760

[45] Goyal L Saha SK Liu LY Siravegna G Leshchiner I Ahronian LG . Polyclonal secondary fgfr2 mutations drive acquired resistance to fgfr inhibition in patients with fgfr2 fusion-positive cholangiocarcinoma. Cancer Discov. (2017) 7:252–63. doi: 10.1158/2159-8290.Cd-16-1000 28034880 PMC5433349

[46] Branch of Biliary Surgery, Chinese Society of Surgery, Chinese Medical Association, Working Group of Biliary Surgeons, Chinese College of Surgeons, Chinese Medical Doctor Association . Expert consensus on conversion therapy of biliary tract cancer (2025). Zhonghua Wai Ke Za Zhi. (2025) 63:453–60. doi: 10.3760/cma.j.cn112139-20250310-00118 40258785

[47] Dong X Zhang Z Zhang Q Chen L Cao G Liu C . Triple therapy in biliary tract cancers: Gemox plus immune checkpoint inhibitor in combination with lenvatinib or ngs-guided targeted therapy. J Cancer Res Clin Oncol. (2023) 149:1917–27. doi: 10.1007/s00432-022-04166-z 35802197 PMC11797449

[48] Zhang T Zhu C Zhang N Zhang L Wang S Xun Z . Lenvatinib combined with pd-1 inhibitor plus gemox chemotherapy versus plus haic for advanced biliary tract cancer. Int Immunopharmacol. (2024) 129:111642. doi: 10.1016/j.intimp.2024.111642 38325044

[49] Li S Yu G Wang M Feng S Wang S Piao M . Efficacy and safety of local-regional therapy combined with chemotherapy, immune checkpoint inhibitors and lenvatinib as first-line treatment in advanced intrahepatic cholangiocarcinoma: A multicenter retrospective cohort study. Cancer Immunol Immunother. (2025) 74:229. doi: 10.1007/s00262-025-04085-1 40445365 PMC12125452

